# Post-traumatic osteoid osteoma in an 18-year-old adolescent

**DOI:** 10.1259/bjrcr.20150141

**Published:** 2015-06-29

**Authors:** E Vancamp, F M Vanhoenacker, G Vanderschueren

**Affiliations:** ^1^Department of Radiology, Antwerp University Hospital, Edegem, Belgium; ^2^Department of Radiology, Sint-Maarten General Hospital, Duffel-Mechelen, Belgium; ^3^Department of Radiology, Ghent University Hospital, Ghent, Belgium; ^4^Department of Radiology, University Hospital Gasthuisberg, Leuven, Belgium

## Abstract

Osteoid osteoma (OO) is a painful, benign bone-forming lesion, which often poses a diagnostic challenge. The aetiology of OO is still poorly understood. Although not generally accepted, an association with previous trauma or infection has occasionally been suggested. We present a case of an OO 12 years following an ulnar fracture. Radiologists should consider OO as a potential delayed “complication” of a previous fracture. Persistent pain at a previous fracture site should alert the clinician to request cross-sectional imaging. CT scanning plays a pivotal role in the correct diagnosis of OO.

## Summary

Diagnosing osteoid osteoma (OO) can be a significant challenge owing to its ambiguous presentation and unclear aetiology. This article describes a case of OO at the site of a previous ulnar fracture, sustained 12 years previously. The patient experienced nocturnal pain at the fracture site, which was relieved by salicylates. The final diagnosis was primarily based on imaging findings. Performing a CT scan is mandatory when OO is suspected. Although the association between OO and previous trauma remains controversial, persistent pain at a previous fracture site should raise the index of clinical suspicion of OO as a post-traumatic “complication”. Internal fixation may be the most important predisposing factor in this process.

## Case presentation

**Figure 1.**
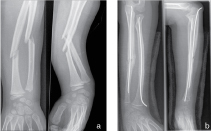
(a) Initial radiograph (posteroanterior film) 12 years previously shows an oblique fracture at the mid-diaphysis of both radius and ulna. (b) Postoperative anteroposterior film depicting intramedullary pinning and beginning signs of callus formation. The radius demonstrates nearly complete bony bridging. The ulna shows partial but incomplete bony bridging.

**Figure 2.**
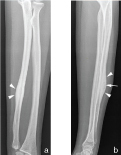
Anteroposterior (a) and lateral (b) plain radiographs at current admission reveal a focal intracortical lucency (white arrow) adjacent to the previous fracture site with surrounding sclerosis and cortical thickening (white arrowheads).

**Figure 3.**
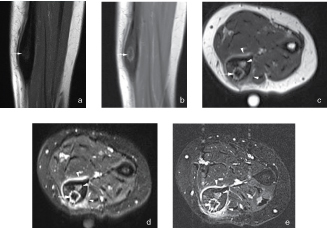
(a) Coronal *T*_1_ weighted image. (b) Coronal *T*_2_ weighted image. (c) Axial *T*_2_ weighted image. (d) Axial fat-suppressed *T*_1_ weighted image after administration of gadolinium contrast. (e) Axial subtraction of the images before and after gadolinium contrast administration. MRI shows a focal intracortical lesion in the ulna with low central signal intensity and mineralization on both *T*_1_ and *T*_2_ weighted images (white arrows). After administration of gadolinium contrast, there is marked peripheral enhancement of the lesion with perilesional bone marrow and soft-tissue oedema (white arrowheads).

**Figure 4.**
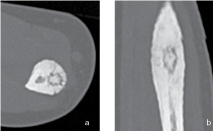
(a) Axial CT image. (b) Coronal reformatted CT image. CT visualization of a cortical nidus in the ulna, suggestive of an osteoid osteoma.

An 18-year-old female was referred to our institution complaining of pain in the left mid-forearm. Previous medical history included a fracture of both radius and ulna 12 years previously, which had been successfully treated with reduction and by intramedullary pinning (Figure 1). Several months before the current referral, there was an insidious onset of pain, which was gradually increasing, more intense at night and relieved by salicylates. On physical examination, there was moderate swelling at the old fracture site. There were no signs of local or systemic inflammation. Conventional radiographs of the left forearm revealed a lucent area adjacent to the previous fracture site with surrounding sclerosis and cortical thickening (Figure 2). Subsequent MRI showed an oval intracortical lesion in the ulna, with central hypointensity to skeletal muscle on both *T*_1_ and *T*_2_ weighted images. After administration of gadolinium contrast, there was marked peripheral enhancement of the lesion with perilesional bone marrow and soft-tissue oedema (Figure 3). Because imaging characteristics were highly suggestive of an OO, an additional CT scan was performed (Figure 4). This examination showed pathognomonic features of an OO with a central calcified nidus at the site of the previous fracture.

## Clinical presentation

OO is a benign osteoblastic lesion characterized by a core or nidus of osteoid tissue that is surrounded by a zone of reactive bone formation. OOs comprise around 11% of all benign primary bone tumours. They are more prevalent in males and usually occur during the second or third decade of life. Since the lesion is richly innervated by nerve fibres, pain is the most common symptom. The pain is gradually progressive, often more intense at night and typically relieved by the administration of salicylates. Sometimes the pain is referred to adjacent joints or is poorly localized, contributing to the difficulty in diagnosing the tumour. Superficial lesions may present with swelling, tenderness and redness, and therefore may mimic the clinical picture of osteomyelitis. Depending on its location within the bone, the lesion is classified as cortical, medullary (cancellous) or subperiosteal. OO usually occurs in the shaft of the long bones, especially the femur and the tibia.^1^

## Aetiology

The pathogenesis of OO has not yet been fully understood. Whether it represents a true neoplasm, a reactive lesion in response to trauma, inflammation or infection, or an unusual healing or vascularization process is still a matter of debate. Most reports do not mention any aetiological relationship between trauma and formation of OO. More recently, however, some authors have documented OO occurring after traumatic events or fractures. Our review of the literature has identified seven cases of OO as a delayed sequelae of a sustained fracture in adolescents. The predominantly affected bone was the tibia, followed by the femur and the radius, with pain presenting between 2 and 8 years after trauma.^2–8^ In five cases, the fracture was reduced and treated by internal fixation. It has been suggested that invagination of the periosteum during fracture, reduction or pinning may act as a predisposing factor for the development of OO,^3^ which is also in line with the observations in our case. However, since no scientific study has proven this hypothesis so far, the association between traumatic events and OO remains doubtful.

## Imaging findings

On plain radiographs, an OO typically presents as a round or oval intracortical radiolucent focus representing the nidus that contains a variable amount of central mineralization, accompanied by reactive sclerosis and cortical thickening. Extensive sclerosis may obscure nidus visualization on plain radiographs. CT scanning is much more accurate in detecting the nidus in the sclerotic area. The nidus has a low attenuation, although a central area of high attenuation, representing mineralized osteoid, is often seen. Surrounding reactive sclerosis ranges from mild sclerosis of the cortex to extensive periosteal reaction and new bone formation. On MRI, the nidus is of low-to-intermediate signal intensity on both *T*_1_ and *T*_2_ weighted images, depending on the amount of cortical mineralization. Administration of gadolinium-based contrast material may demonstrate strong enhancement of the nidus. Oedema in the adjacent bone marrow and soft tissue may also be seen on *T*_2_ weighted images and on contrast-enhanced images. MRI may be non-specific and may mimic other diseases (e.g. stress fracture or osteomyelitis) if extensive surrounding bone marrow oedema obscures the nidus.^1^

## Differential diagnosis

The main differential diagnosis includes cortical osteitis and longitudinal stress fracture. Septic cortical osteitis is a rare subgroup of bone infection caused by haematogenous spread that is predominantly or exclusively located in the cortex of long tubular bones. It usually affects adolescents and young adults. Plain radiographs may reveal focal cortical osteolysis along with the long axis of the bone with a central linear density and limited periosteal reaction. CT scanning is the preferred technique to detect an intracortical sequestrum of dead bone resulting in the “cortical split sign”. Unlike the osteolysis in cortical osteitis, the lucent area in OO is smaller and has more smooth, round margins. A longitudinal stress fracture can also cause an intracortical lucency, which has a more linear shape compared with an oval or rounded shape in OO.^1,9^

## Treatment

OOs are known to be self-limiting tumours that can be treated conservatively with salicylates. However, the response to salicylates is variable and most patients are unable to continue the treatment regimen because of persistent pain. Surgical excision is not always straightforward because of the inherent inability to locate the nidus during surgery. Furthermore, removal of larger amounts of bone is associated with a risk of fracture. Therefore, most OOs are currently treated by CT-guided thermocoagulation or radiofrequency ablation (RFA).^10^ In our case, CT-guided percutaneous curettage was performed, followed by RFA procedure. The patient recovered soon and at the 6-month follow-up, she was completely pain free.

## Learning points

1. Unexplained post-traumatic pain at a fracture site warrants further investigation by imaging.
2. CT scan is the preferred technique for detection and characterization of OO.
3. The exact pathogenesis of OO remains a matter of debate.
4. Although a post-traumatic aetiology has been suggested in the literature, this is not generally accepted as the main pathogenic mechanism.
5. Based on the existing literature, internal fixation of the fracture may be a predisposing factor for development of an OO several years following trauma.
